# NRF2 in age-related musculoskeletal diseases: Role and treatment prospects

**DOI:** 10.1016/j.gendis.2023.101180

**Published:** 2023-11-27

**Authors:** Xiangyu Zhang, Hengzhen Li, Lin Chen, Yuxiang Wu, Yusheng Li

**Affiliations:** a Department of Orthopedics, Xiangya Hospital, Central South University, Changsha, Hunan 410008, China; b National Clinical Research Center for Geriatric Disorders, Xiangya Hospital, Central South University, Changsha, Hunan 410008, China; c Department of Health and Physical Education, Jianghan University, Wuhan, Hubei 430056, China

**Keywords:** Intervertebral disc degeneration, NRF2, Osteoarthritis, Osteoporosis, Sarcopenia

## Abstract

The NRF2 pathway is a metabolic- and redox-sensitive signaling axis in which the transcription factor controls the expression of a multitude of genes that enable cells to survive environmental stressors, such as oxidative stress, mainly by inducing the expression of cytoprotective genes. Basal NRF2 levels are maintained under normal physiological conditions, but when exposed to oxidative stress, cells activate the NRF2 pathway, which is crucial for supporting cell survival. Recently, the NRF2 pathway has been found to have novel functions in metabolic regulation and interplay with other signaling pathways, offering novel insights into the treatment of various diseases. Numerous studies have shown that targeting its pathway can effectively investigate the development and progression of age-related musculoskeletal diseases, such as sarcopenia, osteoporosis, osteoarthritis, and intervertebral disc degeneration. Appropriate regulation of the NRF2 pathway flux holds promise as a means to improve musculoskeletal function, thereby providing a new avenue for drug treatment of age-related musculoskeletal diseases in clinical settings. The review summarized an overview of the relationship between NRF2 and cellular processes such as oxidative stress, apoptosis, inflammation, mitochondrial dysfunction, ferroptosis, and autophagy, and explores the potential of targeted NRF2 regulation in the treatment of age-related musculoskeletal diseases.

## Introduction

Musculoskeletal diseases (MSDs) are injuries to the muscles, bones, joints, and adjacent connective tissues that result in temporary or lifelong limitations in function and participation. Globally, 1.71 billion people have MSDs, severely limiting mobility and flexibility, leading to early retirement, reduced quality of life, and reduced ability to participate in social activities.^1^ The number of people with MSDs rapidly increases due to population growth and aging. The latest survey shows that approximately three-quarters of people over 65 suffer from MSDs.^2^ The cost of MSDs has become an enormous burden on our society and health care system. With age, the loss of physical function and skeletal muscle mass poses a significant threat to later loss of independence. This brings attention to the social burden of age-related musculoskeletal diseases (AMSDs), which account for the majority of MSDs. AMSDs such as osteoarthritis (OA), osteoporosis (OP), sarcopenia, and intervertebral disc degeneration (IDD) pose significant challenges to human health.

Aging is the primary risk factor for AMSDs. The abnormal production or accumulation of reactive oxygen species (ROS) has long been associated with aging.^3^ One of the key players in the evolutionarily conserved cellular defense mechanism against oxidative stress is nuclear factor (erythroid-derived 2)-like 2 (NRF2), which is a Cap'n'Collar basic leucine zipper transcription factor consisting of seven NRF2-ECH homology (Neh) domains.^4^ Under non-stress conditions, the protein level of NRF2 always remains low in order to maintain oxidative homeostasis. When oxidative or exogenous stress is exposed to cells, the inhibitor complexes of NRF2 are dissociated, allowing NRF2 to bind to antioxidant response elements (AREs) by forming a heterodimer with small musculoaponeurotic fibrosarcoma (sMAF) proteins. This results in the activation of cell-protective genes containing AREs, which stimulates the activity of antioxidant defense components like heme oxygenase-1 (HO-1), glutathione peroxidase, superoxide dismutase, NAD(P)H:quinone oxidoreductase 1 (NQO1), thioredoxin reductase, and ferritin.^5^^,^^6^ Consequently, NRF2 can regulate the expression of antioxidants to safeguard cells and delay senescence. However, NRF2 expression declines with age, causing an age-dependent accumulation of ROS, which in turn contributes to persistent chronic inflammation,^7^ imbalance of bone homeostasis,^8^ mitochondrial damage of skeletal muscle,^9^ and other factors, thereby increasing the risk of AMSDs.

## NRF2 signaling pathway

### The structure of NRF2

NRF2 belongs to the Cap'n'Collar subfamily of basic leucine zipper factors, which consists of nuclear erythroid 2 and related factors (NRF1, NRF2, NRF3), and two BTB (broad complex-Tramtrack-Bric-a-brac domain) and Cap'n'Collar homologous proteins (Bach1, Bach2).^10^ NRF2 consists of seven Neh domains with different functions. Because the Neh1 domain binds to the sMAF protein, NRF2 can attach to DNA containing AREs.^11^^,^^12^ The Neh2 domain can recruit Keap1, a dimeric redox-sensitive substrate adaptor.^13^ This promotes the binding of NRF2 to Keap1 and prevents NRF2 from initiating its antioxidant effect. The Neh3 domain transactivates NRF2 to promote the regulation of transcription by AREs. This structure is closely related to transcriptional activity.^14^ The Neh4 and Neh5 domains of NRF2 are responsible for recruiting transcriptional coactivators such as cAMP response element-binding protein-binding protein or receptor-associated coactivator 3, allowing for transcriptional activation or inhibition.^15^ Meanwhile, the Neh6 domain mediates the NRF2 degradation pathway independent of Keap1.^13^ The binding of the Neh7 domain to retinol inhibits NRF2-ARE-dependent gene expression.^16^

### The Keap1-NRF2-ARE pathway and its activation

Keap1 is a receptor that specifically binds to NRF2 and is mainly located in the cytoplasm. Five domains (NTR, BTB, IVR, DGR, and CTR) comprise the keap1, which plays a critical role in the ubiquitination degradation of NRF2, both in the absence and presence of oxidative stress.^17^^,^^18^

Downstream of NRF2 are mainly AREs, also known as electrophilic reaction elements. AREs are cis-reactive elements that encode the promoter region of many detoxifying enzymes and cell protection proteins.^19^ Under oxidative stress, dissociated NRF2 translocates into the nucleus, forming a heterodimer with sMAF. The NRF2-sMAF heterodimer is recruited into this sequence and then specifically binds to AREs to initiate downstream target genes.^20^

The NRF2-ARE signaling pathway is tightly controlled by the activity of NRF2 and its regulation at the protein level, including its synthesis and degradation. In normal cells, the Neh2 domain of NRF2 is bound by the DGR region of Keap1, which retains NRF2 in the cytoplasm. Keap1 acts as a substrate adapter that targets NRF2 for ubiquitination and delivers it to the 26S proteasome for degradation.^21^ However, during times of stress, Keap1 undergoes conformational changes due to interaction with electrophilic compounds or oxidants, resulting in the dissociation of Keap1 from NRF2 and allowing the accumulation of newly synthesized NRF2.^22^ The accumulation of NRF2 triggers its activation and translocation into the nucleus, and then it forms a heterodimer with sMAF and recognizes AREs, leading to the expression of a range of downstream protective genes, including antioxidant enzymes and phase II detoxification enzymes. This signaling pathway enhances the antioxidant and detoxification capacity of cells and thus has a crucial protective role.^23^

### Other regulation mechanisms of NRF2

The transcription of NRF2 can be modulated by multiple signaling pathways, which can activate or inhibit the Keap1-CUL3-RBX1 complex, SCF/β-TrCP complex, or HRD1, thereby regulating NRF2 protein levels. It has been observed that various mechanisms, including competitive binding of proteins containing the ETGE motif, oxidative modification of cysteine residues, increased levels of CUL3-RBX1 E3 ligase inhibitors, mTOR, p62/sequestosome 1, and protein–protein interactions, can interfere with the interaction between NRF2 and Keap1, leading to NRF2 accumulation.^24^ Correspondingly, increased methylation of the Keap1 promoter has been shown to up-regulate NRF2 expression. Moreover, the interaction between it and SCF/β-TrCP can be regulated by various inhibitors, such as GsK3-β, mTOR, PI3K-AKT-PKC, insulin/growth factor, and extracellular signal-regulated kinase (ERK)/p38-mitogen-activated protein kinases (MAPK).^25^^,^^26^ HRD1 inhibitors can also prevent endoplasmic reticulum stress-related NRF2 degradation.^27^ The transcriptional regulators Bach1 and c-Myc are transcription inhibitors of AREs. Under normal conditions, Bach1 binds to the sMAF protein to form a heterodimer, inhibiting its recognition of sMAF and preventing NRF2 from binding to it. A higher heme level inhibits Bach1 binding activity.^28^ The c-Myc protein has been shown to interact with the ARE binding complex and modulate the NRF2-ARE signaling pathway. This interaction can result in an increased rate of its degradation.^29^ Interestingly, both Bach1 and c-Myc increase during the aging process.^30^

### Basic functions of NRF2

NRF2 exerts a crucial function in human physiological regulation by participating in a multitude of processes, including, but not limited to, redox homeostasis, metabolic pathways, DNA damage repair, protein quality control, apoptosis prevention, inflammation, mitochondrial function, and regulation of phase I, II, and III drug/xenobiotic metabolism, as well as iron/heme metabolism.^13^^,^^31^, ^32^, ^33^

### NRF2 and metabolism

NRF2 is involved in the regulation of numerous metabolic genes, such as those associated with purine nucleotide and serine biosynthesis, as well as the pentose phosphate pathway. Additionally, NRF2 is known to activate genes that promote mitochondrial function and fatty acid metabolism. The activation of NRF2 increases glucose intake, up-regulates the glycolysis and pentose phosphate pathways, and improves the efficiency of fatty acid oxidation.^31^

### NRF2 and cell senescence

In general, NRF2 has been shown to have a preventative role in cell senescence and apoptosis. Studies have demonstrated a decline in NRF2 expression in aging cells compared with younger cells, particularly in fibroblasts.^34^^,^^35^ As older fibroblasts are more vulnerable to the effects of oxidative stress, the down-regulation of NRF2 expression may be implicated in various age-related diseases.^25^ Research findings indicate that activation of NRF2 by its inducer can stimulate the production of proteasomes by activating AREs situated on the nearby promoter regions of various proteasome subunits. To put it another way, the activation of NRF2 has been shown to up-regulate the expression of proteasome subunits via AREs. The proteasome is closely related to cell senescence and can extend survival, enhance cell protection, prevent senescence, and even reverse the senescence phenotype and restore proliferation.^36^ Further investigation into the relationship between NRF2 and cell aging has revealed a correlation between NRF2 and telomerase reverse transcriptase. Studies indicate that elevated NRF2 levels lead to an increase in telomerase reverse transcriptase expression, a crucial component of the telomerase complex that facilitates the elongation of terminal DNA telomeres.^32^ This results in continued cell division prevents chromosomal degeneration and helps prevent cell senescence and apoptosis.^37^ Conversely, decreased NRF2 levels increase the likelihood of cell senescence and apoptosis.

### NRF2 and inflammatory

NRF2 has been shown to possess anti-inflammatory effects through multiple pathways. Several recent studies have demonstrated a crosstalk between the NRF2-ARE system and the expression of proinflammatory mediators, as well as with the macrophage metabolism and nuclear transcription factor-kappa B (NF-κB) pathway.^38^ The regulation of the oxidative defense system is thought to be mainly achieved through NRF2-sMAF heterodimer binding with AREs to achieve anti-inflammatory effects. For example, its mediated HO-1 up-regulation affects the intracellular redox signal and significantly reduces inflammation.^39^ Crosstalk with the NF-κB pathway may be the main reason for the anti-inflammatory effect of NRF2 during inflammation. NF-κB is competitively inhibited by NRF2 because both factors compete to bind to cAMP response element-binding protein-binding protein, a cofactor required for both transcriptional reactions. When the activity of NRF2 increases, the binding ability of NF-κB to cAMP response element-binding protein-binding protein is inhibited, thereby reducing the expression level of inflammation-related genes.^40^ In the case of inflammation, NRF2 also has another mechanism with NF-κB. Studies have shown that up-regulation of NRF2 can enhance the clearance of α-synuclein, which is a proinflammatory protein promoting the activation of NF-κB, to reduce inflammation.^41^ Furthermore, its mediated antioxidant proteins such as NQO1, HO-1, or glutamate cysteine ligase inhibit NF-κB activation.^38^ These studies suggest a potential interplay between NRF2 and NF-κB. An intriguing finding is that NRF2 can directly interact with DNA in the proximity of the IL-1β and IL-6 genes, leading to the inhibition of RNA polymerase II binding to transcription complexes. This results in the regulation of proinflammatory cytokine expression without affecting the recruitment of NF-κB p65, and notably, is not dependent on ARE-mediated signaling.^33^

### NRF2 and mitochondria

NRF2 is significantly associated with mitochondrial function. Age-related decline in it may be a critical driver of mitochondrial dysfunction associated with degenerative diseases. Some studies have shown that the activation of NRF2 offsets the increase in ROS in mitochondria and maintains the structural integrity of mitochondria through the transcriptional up-regulation of uncoupling protein 3.^42^ NRF2 is also associated with a variety of cell death modes via mitochondria. It regulates the expression of anti-apoptotic mitochondrial proteins, including BCL-2 and BCL-xL, which are critical in the prevention of cell death by maintaining mitochondrial integrity and regulating the release of apoptogenic factors.^43^ If these proteins are expressed at low levels, a proapoptotic cascade can occur. For instance, increased activation of mitochondrial permeability transition pores damages the tricarboxylic acid cycle and reduces the production of ATP. The enhanced generation of hydroxyl radicals and superoxide, as well as the translocation of cytochrome c into the cytoplasm, trigger irreversible damage and impairment of mitochondrial activity. The release of cytochrome c stimulates the assembly of apoptotic bodies, culminating in apoptosis. Accordingly, reducing the activity of NRF2 results in an increased vulnerability to apoptosis. Inhibition of its activity can lead to decreased permeability transition pores, which can help preserve mitochondrial function, ultimately mitigating age-associated mitochondrial dysfunction.^44^

### NRF2 and ferroptosis

Ferroptosis results from iron-dependent and ROS-dependent oxidative damage leading to cell death through lipid peroxide accumulation. NRF2 maintains iron/heme homeostasis by regulating ferritin, ferroportin, and HO-1.^45^^,^^46^ The cystine/glutamate transporter xCT and glutathione peroxidase 4, the antioxidant system of ferroptosis, are established as NRF2 transcriptional targets.^47^ It is involved in almost all aspects of ferroptosis. NRF2 is a critical mitigator of lipid peroxidation and ferroptosis. NRF2 activated by sulforaphane is AMPK-dependent and inhibits cardiac cell ferroptosis by up-regulating ferritin and xCT levels.^48^ On the other hand, some studies indicate that NRF2 may also promote ferroptosis. In particular, a recent study revealed that its mediated induction of HO-1 may lead to the release of free iron during the progression of cardiomyopathy, thereby contributing to ferroptosis.^49^

### NRF2 and autophagy

The NRF2 signaling pathway is thought to be involved in various autophagy processes, including mitophagy. The NRF2 and autophagy pathways can both respond to stress through a cascade of antioxidant and cellular defense genes. The mitophagy pathway is mainly dependent on Parkin RBR E3 ubiquitin protein ligase, and NRF2 can regulate the expression of Pink1 by binding to the four ARE sequences present in the Pink1 promoter.^50^ Knockdown of Parkin/Pink1 alters proteostasis components and interrupts mitophagy. Mitophagy rates can be reversed by NRF2 activation.^51^ When autophagy is impaired, genes regulated by NRF2-ARE are up-regulated due to Keap1 being separated into aggregates by p62.^52^ The synthesized 1,4-diphenyl-1,2,3-triazole can not only activate NRF2 but also promote mitochondrial autophagy and contribute to mitochondrial homeostasis.^42^ The relationship between autophagy and NRF2 needs to be further elucidated.

### NRF2 and stem cells

Maintaining tissue homeostasis and repair is dependent on the renewal and differentiation of stem cells. However, the decline in stem cell function with age in all tissues and organs is well documented. Numerous studies have shown that NRF2 has an impact on cell proliferation and differentiation (Table 1). As conditions vary, it may have different effects on stem cell regeneration during aging.^53^ One study has reported that increased NRF2 activity promotes self-renewal and inhibits differentiation of human embryonic stem cells by up-regulating proteasome formation.^54^ Much evidence shows that NRF2 activation leads to the proliferation of tissue stem cells.^55^^,^^56^ Nonetheless, excessive activation of it can lead to uncontrolled regeneration and eventually stem cell failure.^54^ These findings underscore the importance of maintaining a balance in NRF2 activity to sustain the viability and function of stem cells and prevent their failure. Moreover, targeting the NRF2 pathway may optimize the regulation of stem cell function with age and avert potential age-related diseases. Utilizing the pivotal role of NRF2 in senescence, apoptosis, and cell differentiation represents a novel strategy. However, implementing this strategy is relatively complex. For example, ROS regulated by NRF2 are considered essential regulators in stem cell self-renewal.^57^ The functions of ROS in stem cells are diverse, as they can regulate processes such as cell proliferation, differentiation, self-renewal, replication, and senescence in pluripotent stem cells by influencing the frequency of the c-MAF gene, which may be related to the environment. In some cases, the increase in ROS, including H_2_O_2_, the primary source of the ROS signaling pathway, stimulates the proliferation of stem cells. However, in other cases, the accumulation of ROS can impair the function and maintenance of stem cells.^58^, ^59^, ^60^ The contribution of it to stem cell activity is different in different studies. For instance, in a study on the effect of age on bone marrow-derived mesenchymal stem cells, inhibition of the NRF2-ARE pathway resulted in decreased proliferation, colony formation, and osteogenic differentiation of bone marrow-derived mesenchymal stem cells in the elderly.^61^ In another study that investigated the effect of silencing NRF2 and autophagy on adipose mesenchymal stem cells, osteoblasts (OBs) were found to be generated from adipose mesenchymal stem cells. The injection of NRF2-inhibited stem cells *in vivo* was observed to promote bone formation.^62^ Furthermore, the activation of the Toll-like receptor 4-mediated NF-κB signaling pathway through antioxidant-driven NRF2 was identified as a possible approach to promote adipose mesenchymal stem cell differentiation.^63^

**Table 1:** Functions of NRF2 in cell proliferation and differentiation.

Cell type	Contribution	Function	Molecular mechanism	Study model	Reference
Osteoclasts	Differentiation	NRF2 deficiency promotes osteoclast differentiation	NRF2 dysfunction promotes osteoclast differentiation through ROS accumulation	RAW 264.7 cells	64
Osteoblasts	Differentiation	NRF2 overexpression inhibits osteoblast differentiation	NRF2 inhibits osteoblast differentiation by binding to RUNX2, which plays an important role in osteoblast generation	MC3T3 cells	65
Chondrocytes	Differentiation	NRF2 overexpression inhibits chondrogenesis	NRF2 activation inhibited chondrogenesis through suppressing autophagy	C3H10T1/2 cells	66
Chondrocytes	Differentiation	NRF2 overexpression inhibits chondrogenesis	NRF2 activation inhibited chondrogenesis by reducing the expression of chondrocyte differentiation markers such as Col II, Col X, and osteopontin	ATDC5 cells	67
Chondrocytes	Differentiation	NRF2 deficiency inhibits chondrogenesis	NRF2 deficiency inhibits the chondrogenesis related gene SOX9	Human C-28/I2 chondrocytes	68
Fibroblast-like synoviocytes	Maintenance	NRF2 knockdown enhances the proliferation and invasion of fibroblast-like synoviocytes in inflammation	NRF2 may control the proliferation and invasion of fibroblast-like synoviocytes induced by TNF-α by regulating JNK activation	NRF2 knockdown *in vitro*	69
Embryonic stem cells	Maintenance	Normal NRF2 activation contributes to the self-renewal of embryonic stem cells and inhibits their differentiation	NRF2 controls proteasome activity by regulating Pomp	NRF2 knockdown *in vitro*	54
Hematopoietic stem cells	Maintenance	The activation of NRF2 promotes hematopoietic reconstitution at the early stage after radiation injury	NRF2-mediated Notch signaling improves hematopoietic stem cells' function	Keap1 conditional knockout mice and NRF2 knockout mice	70
Myosatellite cells	Proliferation	NRF2 deficiency delayed stem cell proliferation after ischemia-reperfusion injury	NRF2 directly enhanced MyoD expression	NRF2 knockout mice	55

### Roles of NRF2 in age-related musculoskeletal diseases

Based on the information above, it is evident that NRF2 promotes the expression of multiple genes to initiate a protective response within cells. Its involvement in cell metabolism, antioxidant signaling, protein homeostasis, and iron metabolism is critical. Therefore, the expression of NRF2 plays a vital role in various cellular processes, including oxidative stress, inflammation, mitochondrial dysfunction, cell senescence, apoptosis, proliferation, and differentiation. Studies on the musculoskeletal system reveal a strong link between mitochondrial dysfunction, increased ROS, and age-related loss of function. In addition, cell senescence, apoptosis, and inflammation can exacerbate the progression of musculoskeletal degeneration significantly.^71^^,^^72^ Its role in age-related diseases is also noteworthy and may hold promising therapeutic potential.

Therefore, in the next section, we will summarize the studies of NRF2 in four common AMSDs, including sarcopenia, OP, OA, and IDD, and investigate the regulatory mechanisms of NRF2 in AMSDs.

## Sarcopenia

### Role of NRF2 in sarcopenia

Sarcopenia is a degenerative and systemic condition that results in the loss of skeletal muscle mass and function, especially in the elderly population.^73^ According to the criteria established by the European Working Group on Sarcopenia in Older People, the prevalence of sarcopenia in the UK among men and women aged 85 and above is estimated to be 3.6%.^74^ Most age-related endogenous factors, such as loss of neuromuscular function, inflammation, and hormonal abnormalities, as well as exogenous factors, such as sedentary behavior and malnutrition, can contribute to the progression of sarcopenia.^75^ The occurrence of sarcopenia in older individuals may also be influenced by genetic factors.^76^^,^^77^

The underlying causes of sarcopenia are multifactorial and include various mechanisms, including malnutrition, obesity, inflammation, oxidative stress, mitochondrial dysfunction, and apoptosis.^78^, ^79^, ^80^, ^81^ Multiple investigations have demonstrated that the impairment of the NRF2-ARE pathway is linked to muscle atrophy. This process promotes cellular degradation pathways, induces ubiquitination and proapoptotic signals, and affects antioxidant mechanisms and muscle regeneration, especially in the context of aging skeletal muscle.^82^^,^^83^ It should be noted that sarcopenia is characterized not only by a reduction in muscle mass but also by a decline in muscle function. However, there is a limited amount of research investigating the connection between NRF2 and muscle function. One article showed that a lack of NRF2 transcriptional activity in muscle injury does not affect muscle function.^84^ Several lines of evidence suggest that this hypothesis may be incorrect and that NRF2 deficiency significantly aggravates mitochondrial dysfunction and muscle dysfunction in elderly mice and has little effect on young mice.^9^ The decline in NRF2 and the mass function of muscle may be related to age. In addition, the decline of muscle mass and function in old age may be driven by the dysregulation of cellular processes such as autophagy, which can be triggered by ROS accumulation and the dysregulation of the NRF2 pathway. This may lead to increased autophagic flux and muscle damage, which could be a potential mechanism underlying the development of sarcopenia.^85^

Emerging research has provided evidence that the up-regulation of NRF2/HO-1 expression exerts inhibitory effects on skeletal muscle cell death, effectively counteracting skeletal muscle atrophy and fibrosis.^86^^,^^87^ Furthermore, skeletal muscle-specific stem cells are modulated by the family of myogenic regulatory factors to entity triggers and govern the process of repair following injury, thus outlining the process of muscle regeneration. NRF2 has been reported to be a critical pathway for skeletal muscle regeneration. Research findings have revealed that NRF2 expression is higher in regenerated myoblasts compared with its lower expression in healthy muscle fibers.^55^^,^^88^ Numerous studies have demonstrated the critical role of the NRF2 system in the proliferation, differentiation, and activation of skeletal muscle-specific stem cells. Additionally, a recent study has revealed a new insight, showing that the depletion of transferrin receptor 1 in skeletal muscle leads to the exhaustion of skeletal muscle-specific stem cells. This process is accompanied by a reduction in NRF2 protein levels, which disrupts lipid and iron metabolism, ultimately inducing ferroptosis.^89^ Mechanistically, NRF2 has been found to promote muscle regeneration by prolonging the proliferation of skeletal muscle-specific stem cells via the up-regulation of MyoD and inhibiting their differentiation via the down-regulation of myogenin.^55^ In addition, its activation, especially the up-regulation of HO-1 expression, prevents oxidative damage to tendon stem cells and significantly improves tendon formation and matrix regeneration during tendon healing.^90^^,^^91^

**Figure 1:**
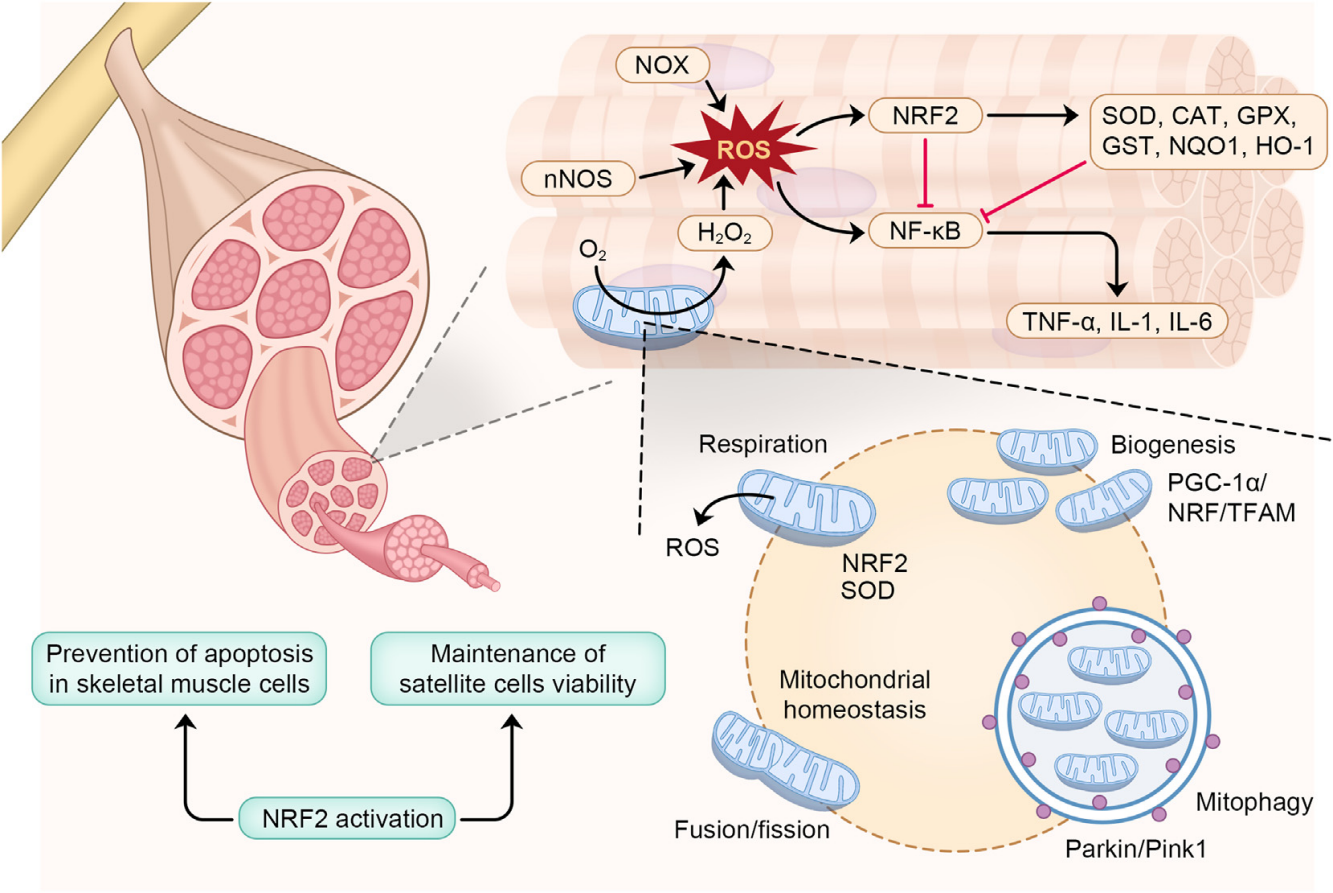
NRF2 is involved in skeletal muscle homeostasis.

Recent studies have provided mounting evidence for the significant role of mitochondrial dysfunction in the development of sarcopenia.^92^ To maintain healthy mitochondria in skeletal muscle cells, the processes of mitochondrial biogenesis, mitochondrial dynamics function, and mitophagy work together.^93^ Mitochondrial biogenesis refers to the synthesis of new mitochondria to replace damaged ones. The fusion/fission mechanisms can repair mild mitochondrial damage, while mitophagy removes irreversibly damaged mitochondria. Any disturbance in these systems can increase the risk of tissue dysfunction and degeneration, ultimately resulting in muscle weakness and sarcopenia.^93^^,^^94^ NRF2 is a mitochondrial biogenesis gene in skeletal muscle (Fig. 1). Its expression can maintain mitochondrial function.^95^ According to the study conducted by Gumeni et al, increased ROS and neurodegeneration were critical features of sarcopenia. Additionally, muscle atrophy and remodeling of the neuromuscular junction are common occurrences in sarcopenia. NRF2 activation in the degenerative neuromuscular phenotype mediated by Parkin/Pink1 knockdown restored mitochondrial function, increased the rate of mitophagy, and significantly alleviated the degenerative neuromuscular phenotype.^51^ Peroxisome proliferator-activated receptor gamma coactivator-1α (PGC-1α) is one of the main proteins that prevent muscle destruction by improving neuromuscular junction structure and delaying the transition from slow to fast fiber type in the target muscle.^96^ Regulatory factors, including PGC-1α, along with downstream transcription factors like NRF2, NRF1, and mitochondrial transcription factor A (TFAM), play a crucial role in the process of mitochondrial biogenesis.^97^ Once activated by phosphorylation or deacetylation, PGC-1α activates NRF2, NRF1, and subsequently TFAM. The synthesis of mitochondrial DNA and protein and the generation of new mitochondria benefit from the activation of the PGC-1α-NRF-TFAM pathway.^98^

### NRF2 activation maintains skeletal muscle and mitochondrial health

The decrease in NRF2 transcriptional activity is one of the driving forces of sarcopenia, and up-regulation of NRF2 expression helps to maintain mitochondrial function and skeletal muscle mass and function. Therefore, up-regulation of NRF2 activity could be a potential way to treat sarcopenia. Antioxidants can activate it to slow the progression of sarcopenia by reducing oxidative stress in skeletal muscle, regulating the proliferation and differentiation of skeletal muscle-specific stem cells, restoring mitochondrial function, and promoting the expression of downstream target proteases. Recently, Blottner et al reported some benefits of treating healthy male subjects with antioxidant cocktails (polyphenols, selenium, vitamin E, and omega-3) in long-term bedrest. Experimental results showed that NRF2-ARE signaling pathway response was enhanced and skeletal muscle degeneration stopped.^99^ Sulforaphane is one of the most representative NRF2 activators, restoring multiple cellular defenses, senescent cell mitochondrial function, glucose tolerance, and skeletal muscle stem cell activation/differentiation through multiple activities of the Keap1-NRF2 signaling, such as mitochondrial biosynthesis, glutathione biosynthesis, and autophagy, during AMSDs. Opposite experimental results were obtained in the case of NRF2 deficiency.^100^^,^^101^ In the experiment on oxidative stress-induced skeletal muscle cell death, the administration of Hirudin resulted in the lowest level of oxidative stress and histological damage at a specific dose of 8 atomic mass units per kg. The treatment with Hirudin also led to the up-regulation of NRF2 and HO-1 levels. Moreover, the treatment significantly reduced the inflammatory response, as evidenced by decreased levels of cytokines such as IL-6, IL-1β, and TNF-α.^87^ Ginsenoside Rb1 is a compound that activates AMPK and subsequently activates the Akt-NRF2 pathway, leading to a reduction in oxidative stress in aged skeletal muscle, induction of myogenic transformation of fibroblasts, and up-regulation of myotube growth and myogenic differentiation.^102^ Conjugated linoleic acid was found to decrease oxidative stress and skeletal muscle oxidative damage, while also up-regulating the expression of NQO1, which is a target gene of NRF2.^103^ Similarly, administration of *Moringa oleifera* leaf extract increased the protein levels of HO-1 induced by NRF2, leading to improved redox homeostasis of glutathione and activities of antioxidant enzymes such as glutathione peroxidase, superoxide dismutase, glutathione S-transferase, and catalase.^104^ In addition, older male rats on a long-term curcumin diet had higher NRF2 nuclear levels and lower oxidative protein damage, with benefits for muscle mass, especially strength.^105^

Certain antioxidants may have additional benefits in sarcopenia caused by certain specific factors. Skeletal muscle atrophy can be induced by denervation caused by aging, drug intervention, peripheral nerve injury, or other diseases, which may result in impaired muscle function.^106^ Isoquercitrin showed a protective effect on muscles at this time, effectively alleviating muscle mass loss after denervation and significantly inhibiting the overexpression of muscle-specific ubiquitin ligase muscle atrophy F-box and muscle ring finger 1 induced by denervation. Myosin heavy chain degradation was reduced in the target muscle. Cytoprotective factors (HO-1, NQO1, and superoxide dismutase 1/2) in muscle were up-regulated, which reduced ROS production, suppressing mitochondrial vacuolation and mitophagy, and delaying the conversion process from slow fibers to fast fibers in the target muscle by triggering PGC-1α expression. In addition, the administration of isoquercitrin deactivated the JAK/STAT3 signaling pathway and reduced the levels of inflammatory cytokines such as IL-6, IL-1β, and TNF-α in the affected muscles.^107^ It is well-established that obesity has a detrimental effect on skeletal muscle mass and function, exacerbating the negative impact of sarcopenia in older individuals, ultimately resulting in sarcopenic obesity.^108^ Quercetin, also a flavonoid, inhibits TNF-α-induced atrophy factors such as the expression of muscle ring finger 1 and muscle atrophy F-box in myotubes and increases nuclear translocation of NRF2 with inactivation of NF-κB while enhancing HO-1 protein levels to prevent muscle atrophy under obesity conditions.^109^ Endogenous substances are also likely to offset the decline in muscle mass in sarcopenia. FGF19 is an essential regulator of metabolic homeostasis that responds to mitochondrial dysfunction and oxidative stress induced by palmitic acid in C2C12 cells. It can increase the levels of HO-1 and NRF2, as well as mitochondrial biogenesis regulators such as NRF1, PGC-1α, and TFAM.^110^ Testosterone induces enhanced expression of NRF1 and NRF2, which can protect C2C12 muscle cells from apoptosis induced by H_2_O_2_ and can also increase muscle mass and function by stimulating IGF-1 protein synthesis.^111^ The optimal activation criterion for NRF2 requires a balance between beneficial ROS levels needed to maintain physiological signaling and restricted harmful ROS levels to protect tissues from oxidative damage.

Physical exercise is currently the primary intervention for sarcopenia. NRF2 activation has been shown to improve exercise capacity by modulating the muscle antioxidant response, mRNA expression of genes associated with mitochondrial biogenesis, as well as glycogen and fatty acid metabolism.^112^ The pharmacological induction of NRF2 by electrophilic chemicals has further elucidated the significance of its mediated exercise intervention in enhancing exercise capacity and regulating skeletal muscle redox status.^101^^,^^113^ Disruption of NRF2 expression impairs exercise capacity and mitochondrial mass.^95^ In the studies of other tissues, such as mouse cardiomyocytes^114^ and primate vascular endothelium/smooth muscle,^115^ most studies suggest that aging also leads to impaired NRF2 function in skeletal muscle.^82^ Nonetheless, several investigations have examined the impact of aging on the expression of NRF2 and its downstream cytoprotective genes in skeletal muscle, but the results were not consistent.^116^^,^^117^ Recently, an increasing body of literature has highlighted the ability of physical exercise to induce the expression of NRF2 in skeletal muscle.^116^^,^^118^ In a study conducted by Safdar et al, higher expression levels of NRF2 and its downstream proteins were observed in older individuals with active lifestyles compared with sedentary older adults and even younger subjects.^116^ Merry et al's study identified NRF2 as an exercise-induced mitochondrial biogenesis gene in skeletal muscle.^95^ This may also be why lifelong physical exercise reverses age-related skeletal muscle loss.^119^ In addition, much physical training can improve other motor age-related diseases by regulating NRF2, such as the prevention of OP by running via its exercise-induced epigenetic disinhibition.^120^ Physical exercise enhances NRF2 function in elderly skeletal muscle.

## Osteoporosis

### Role of NRF2 in bone homeostasis

Bone homeostasis is the dynamic balance between osteoclasts (OCs) and OBs, which maintains the normal process of bone remodeling. Bone remodeling is the process of OC-mediated removal of damaged or old bone and subsequently replacement with new bone formed by OBs.^121^ Excessive activation of OCs can trigger an imbalance between OBs and OCs, leading to the destruction of homeostasis and osteolytic bone diseases such as OP.^122^ NRF2 has been demonstrated to have a vital role in bone homeostasis regulation. Proper NRF2 flux and a good balance of activity are essential for adequately regulating bone metabolism.

OCs are highly differentiated multinucleated macrophages derived from hematopoietic stem cells. The NRF2-ARE signaling pathway is involved in regulating osteoclastogenesis and activity and can reduce the level of ROS in cells by activating the expression of antioxidant targets, thereby inhibiting the MAPK and PI3K/Akt pathway and inhibiting the differentiation and absorption of OCs.^123^ It can also inhibit the differentiation of OCs by inhibiting NF-kB, c-Fos, and nuclear factor of activated T cells, cytoplasmic 1 (NFATc1).^124^^,^^125^ Overexpression of NRF2 reduces intracellular levels of ROS by up-regulating the expression of cytoprotective enzymes such as NQO1, HO-1, and glutamate cysteine ligase *in vitro* and *in vivo*.^126^ It reduces the number of OCs formed and the expression of OC-related genes, including c-Fos, NFATc1, and tartrate-resistant acid phosphatase, and attenuates bone destruction. Treatment with the NRF2 activator schisandrin A inhibited OC differentiation increased its target gene expression in the wild-type mice and inhibited osteoclastogenesis in bone marrow mononuclear macrophages from NRF2-konckout mice.^127^ In the process of RANKL-induced OC differentiation, RANKL promotes the degradation of NRF2 by up-regulating Keap1, reduces intracellular protective enzymes, and increases ROS, thereby promoting the function and differentiation of OCs. In NRF2-deficient mouse cells, RANKL-mediated activation of MAPK-NFATc1 enhanced the formation ability of actin rings and tartrate-resistant acid phosphatase-positive multinucleated cells with more than ten nuclei, suggesting that NRF2 may negatively regulate RANKL-induced OC differentiation and inhibit actin ring formation and bone resorption.^128^ Furthermore, *in vivo* overexpression of NRF2 locally attenuates RANKL-dependent skull destruction induced by lipopolysaccharides.^64^ These studies indicate that Keap1-NRF2 signaling through the cell protective enzymes regulating intracellular ROS level plays a part in osteoclastogenesis.

OBs are derived from mesenchymal stem cells, which have the ability to differentiate in multiple directions. The effect of NRF2 on OB differentiation and function remains debated and may be influenced by genetic factors, age, sex, and physiological or pathological contexts. Some studies have suggested that NRF2 may exert negative effects on bone formation.^129^ HINOI et al overexpressed it in Mc3T3-E1 OBs and found that it inhibited the expression of RUNX2, an indispensable transcription factor for OB differentiation, thus preventing OB differentiation.^65^ KooK et al observed that radiation exposure exerted a dose-dependent inhibitory effect on the maturation and mineralization of OBs, accompanied by a down-regulation of bone-specific genes including bone sialoprotein, osteopontin, and osteocalcin in Mc3T3-E1 cells. However, the negative impact of radiation on OB differentiation in Mc3T3-E1 cells was significantly alleviated by siRNA-mediated silencing of NRF2, leading to up-regulation of RUNX2 and down-regulation of HO-1 expression levels.^130^ Similarly, Park et al cultured the same cells with NRF2 knockdown and calvarial-derived OBs. OB differentiation and calcification were enhanced. The expression levels of alkaline phosphatase, osteocalcin, and RUNX2 were significantly increased in NRF2-deficient OBs.^124^ These studies indicate that NRF2 negatively regulates OB mineralization and differentiation by binding to RUNX2.

**Figure 2:**
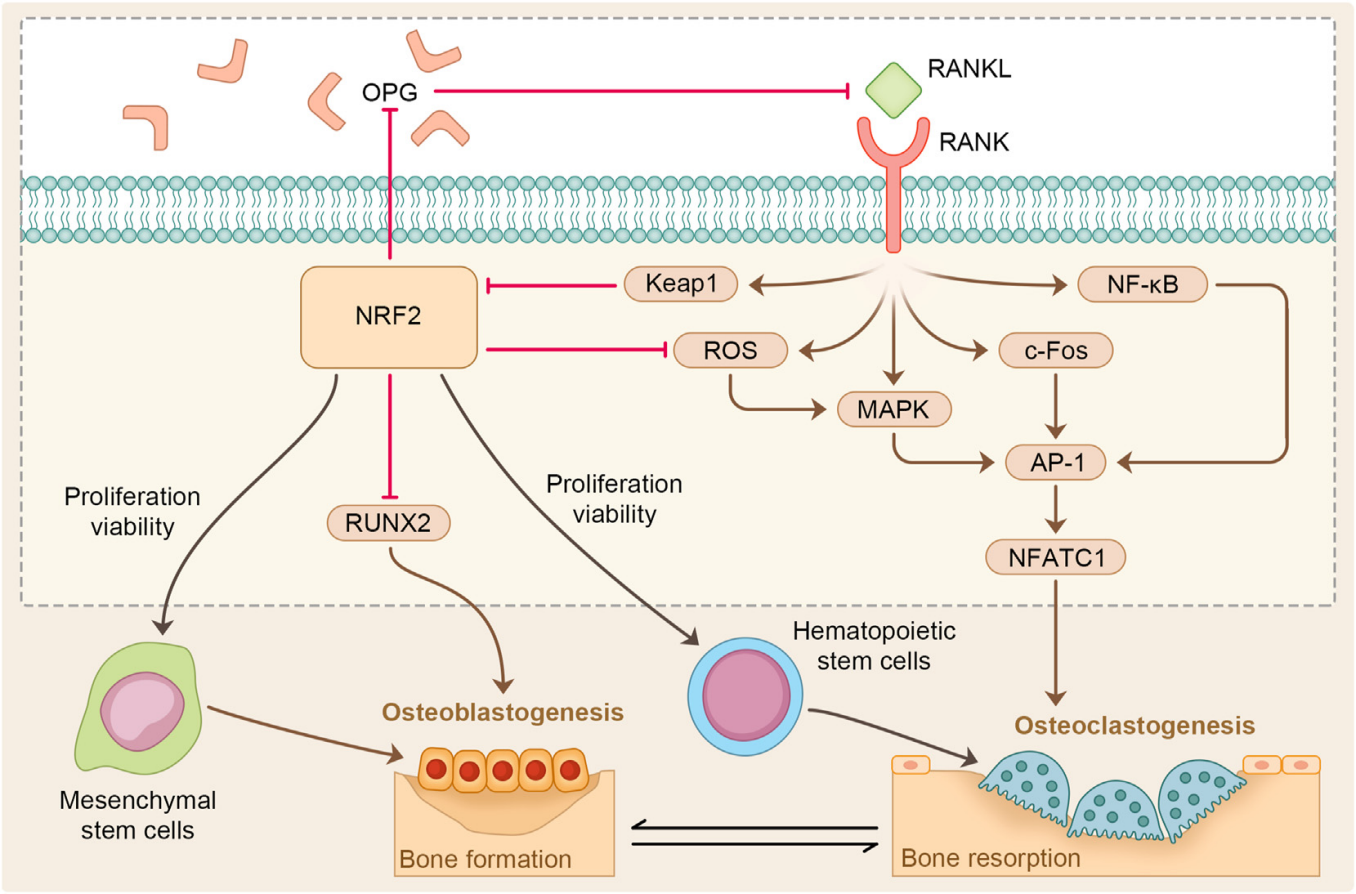
NRF2 controls bone homeostasis.

NRF2 activation inhibited OC and OB differentiation *in vitro*. To explore whether this conclusion holds for bone homeostasis *in vivo*, animal experiments were performed in some studies. The results of Park et al showed that NRF2-deficient mice had increased bone formation compared with wild-type mice. OB number was significantly increased, while OC number and bone resorption parameters were not different. Although inhibition of NRF2 *in vitro* promoted osteoclastogenesis upon RANKL stimulation, there was no significant enhancement of OC function in NRF2-deficient mice. This discrepancy *in vitro* and *in vivo* is due to osteoprotegerin (OPG) secreted by OBs *in vivo*, which is the decoy receptor of RANKL. Osteoclastogenesis is accomplished by a delicate balance of RANKL/OPG. The predominance of OPG inhibits OC differentiation.^131^ NRF2 can directly bind to ARE in the OPG gene promoter region and negatively regulate the expression of OPG in OBs.^124^ Its deficiency may enhance OPG expression, inhibiting OC generation *in vivo* (Fig. 2). Interestingly, an increased bone mass in NRF2 knockout mice is due to increased osteoblastogenesis and bone formation rather than decreased bone resorption. In order to verify the conclusion, YOSHIDA et al established animal models of NRF2 overexpression. Compared with those of control mice, the excessive activation of NRF2 significantly reduced the femur length and bone mass of mice. *In vitro* OB differentiation experiments were performed on newborn mouse skulls. It was observed that the differentiation of mouse OBs was severely impaired.^132^ These results suggest that bone loss in mice is on account of impaired bone homeostasis resulting from reduced OB differentiation, which is inhibited by NRF2 hyperactivation.

However, NRF2 deficiency did not increase bone quality. Some studies have shown that the opposite is true under oxidative stress. The effect of its deficiency was more pronounced in OCs than in OBs. Ischemia-reperfusion generates oxidative stress and can be used as a method to induce an osteoporotic phenotype.^133^ In the study of Rana et al, compared with wild-type mice, NRF2 knockout mice undergoing ischemia-reperfusion had increased bone loss and a more significant number of OCs. Notably, NRF2-deficient OBs showed increased RANKL expression after ischemia-reperfusion, which may account for OC expansion.^134^ The effect of NRF2 deficiency on bone homeostasis *in vivo*, particularly the generation of OCs and OBs, can be perturbed by oxidative stress. Ibáñez et al reached a similar conclusion and found that NRF2 deficiency significantly reduced trabecular bone volume, bone volume fraction, bone surface density, and bone mineral density.^135^ These studies suggest that normal NRF2 activation is required to maintain bone mass. The application of its agonists has a positive impact on the survival of OBs, OCs, and stem cells. NRF2 activation directly suppresses the differentiation of OCs through resisting oxidative stress.

There may be further mechanisms underlying the influence of NRF2 on bone quality. Sánchez et al found that an increase in mitochondrial content during the transition from OBs to osteocytes results in increased ROS levels. NRF2 is activated during osteocytogenesis under oxidative stress conditions and directly transactivates many osteocyte-specific genes, such as Sost, Mepe, and Dmp1. In addition, bone changes in mice lacking NRF2 showed significant sexual dimorphism.^8^ KIM et al found that mice lacking NRF2 exhibited marked impaired bone development and decreased bone mass, thus it is essential for postnatal bone development in normal mice. The results suggest that NRF2 loss impairs bone formation during the early postnatal period. *In vitro* studies showed that the early low bone mass was not caused by any defect in the OBs. Compared with control OBs, primary cranial OBs from NRF2-deficient mice could proliferate and differentiate normally. The impaired postnatal skeletal development in *NRF2*^*−/−*^ mice may be due to a failure of proliferation or viability of the population of osteoprogenitors, which leads to the lower number of OBs observed early in *NRF2*^*−/−*^ mice. Kim et al propose alternative mechanisms, such as FoxO signaling, that can independently activate antioxidant pathways to compensate for the absence of NRF2, indicating the presence of additional pathways exerts a positive influence. This conclusion is validated by the observed recovery of OB number during growth and development.^136^

The exact impact of NRF2 depletion and hyperactivation on bone in animal models remains ambiguous. Nonetheless, the consensus is that NRF2 is crucial in maintaining and acquiring bone mass, and protecting it from various stressors. Many studies have shown that its excessive expression may negatively affect bone formation, whereas moderate activation of NRF2 may enhance bone mass. Interestingly, its impact on bone phenotypes varies by sex, indicating sexual dimorphism. Gender difference in bone phenotypes also suggests that the Keap1-NRF2-ARE pathway, the downstream signal of sex hormone receptors, may be involved. Age and genetic background may be the reasons for the differences in experimental results. NRF2-activated mice also had difficulty excluding the effects of other tissues and cells and Keap1-regulated endocrine systemic factors on bone tissues and cells. Further investigation is necessary to clarify the involvement of NRF2 in cellular differentiation and activity related to bone homeostasis.

### Role of NRF2 in OP

OP is a chronic systemic skeletal disorder characterized by decreased bone mass and microstructural deterioration, leading to an increased susceptibility to fragility fractures. A variety of diseases and treatments can cause or contribute to OP, either as a concomitant primary disease or as a cause of secondary OP.^137^ The risk is higher in elderly individuals, especially in postmenopausal women. With the aging of the population, the social burden caused by OP, especially postmenopausal OP, as the most common clinical type, will further increase.^138^ According to statistics, the global prevalence of OP has risen to the 7th most common and frequently occurring disease, and the specific number of patients has exceeded 200 million.^139^ Bone undergoes continuous renewal to maintain proper integrity and strength, which is controlled by the balance between bone resorption maintained by OCs and bone formation induced by OBs. However, OP is the result of bone resorption of OCs exceeding the bone formation role of OBs. NRF2 is recognized for its vital role in regulating the equilibrium between bone resorption driven by OCs and bone remodeling driven by OBs. A deficiency in NRF2 has been shown to activate NF-кB and c-Fos, leading to increased osteoclastogenesis and promoting RANKL-induced activation of ERK, c-Jun, and p38. Ultimately, this imbalance contributes to the development of OP. This is possibly on account of dysfunction in the production of glutathione and antioxidant enzymes.^128^ NRF2 overactivation enhanced the expression of antioxidant enzymes, inhibited OC differentiation, and attenuated bone destruction.^64^

During the transition from OBs to osteocytes, the number of mitochondria increases due to the higher energy demand. This is followed by an increase in ROS, a mitochondrial byproduct, which NRF2 is activated to remove during osteocytogenesis. The uncontrolled accumulation of ROS in the presence of decreased NRF2 expression due to oxidative stress or aging can lead to an imbalance in bone homeostasis and related diseases, including OP. The study of Sánchez-de-Diego et al showed that mice undergoing oxidative stress express NRF2 activity that directly activates many osteocyte-specific genes, such as Sost, Mepe, and Dmp1. NRF2-deficient mice undergoing ovariectomy exhibited severe osteopenia, and treatment with dimethyl fumarate, an NRF2 activator used in the clinic, restored NRF2 levels and reduced bone loss in an OP model. Additionally, the skeletal phenotype of NRF2-deficient mice was shown to be sexually dimorphic, and the effects of NRF2 deficiency on bone mass were more profound in male mice.^8^ In a diabetic OP mouse model, Yang et al provided evidence that ferroptosis is a significant contributor to osteocyte death. They also found that HO-1 overexpression induced by promoting NRF2-c-JUN interaction in the diabetic microenvironment can exacerbate ferroptosis. The oxidation of heme and the subsequent release of free iron are catalyzed by the enzyme HO-1, which can participate in the Fenton reaction and lead to the generation of ROS that damage mitochondria and cause further release of heme. This vicious cycle ultimately results in the ferroptosis of bone cells.^140^ These findings may seem to contradict the protective role of NRF2 activation in OP, and the involvement of the NRF2/HO-1 axis in ferroptosis is still a matter of debate. However, several studies have reported that up-regulation of NRF2/HO-1 expression can confer protection against ferroptosis by inhibiting ROS accumulation. For example, the ferroptosis inducer RSL3 in microglia and macrophages induces increased NRF2 expression to inhibit cytokine transcription and protect cells from ferroptosis.^141^ In contrast, it has been demonstrated that free iron released during heme degradation through NRF2-mediated HO-1 up-regulation induces cardiac injury.^49^ Excessive up-regulation of HO-1 may be cytotoxic, whereas moderate up-regulation may be cytoprotective.^142^ Differences in tissue and disease models may be responsible for the conclusions. Ma et al rescued osteocyte ferroptosis by melatonin-induced NRF2/HO-1 up-regulation even in the same high glucose-induced diabetic OP model as Yang et al.^143^ Sulforaphane prevents diabetic myocardial ferroptosis via AMPK-mediated NRF2 activation.^48^ It is noteworthy that the activation of NRF2 is mediated by AMPK via sulforaphane, whereas the expression of c-JUN protein is up-regulated and binds to NRF2 in the diabetic OP model. Increased NRF2 expression levels in the OP model and its pharmacologically mediated activation appear to assume diametrically opposing roles in ferroptosis. In conclusion, the increased expression of NRF2 can prevent or even reduce OP, but further studies are needed for more details regarding mechanisms in the disease.

### Moderate NRF2 activation maintains bone mass

Targeted regulation of NRF2 in OP is mainly achieved by inhibiting bone resorption, promoting bone formation, and resisting oxidative stress and cell senescence. Yang's results showed that 1,25-dihydroxyvitamin D reduced NRF2 degradation by suppressing ubiquitin‒proteasome degradation and transcriptionally inhibiting Keap1. Stimulation of this process may lead to the activation of genes targeted by NRF2 transcription, which can then suppress oxidative stress and prevent DNA damage in mesenchymal stem cells derived from bone marrow, while promoting osteogenesis and inhibiting senescence, thereby preventing OP. Oltipraz, an NRF2 degradation inhibitor, could effectively rescue the age-related OP caused by 1,25-dihydroxyvitamin D deficiency.^144^ Briarane-type diterpenoids and schisandrin A suppress RANKL-induced osteoclastogenesis by regulating NRF2.^127^^,^^145^ In addition to inhibiting the formation and bone resorption activities of OCs by resisting oxidative stress through the pharmacologically activated NRF2 pathway, orcinol glucoside also reduces OC autophagy through the interaction between NRF2 and the mTOR signaling pathway.^146^ However, the lncRNA XIST, which is highly expressed in the serum and monocytes of OP patients, inhibits OB differentiation and promotes OP by overactivating NRF2 via targeting CUL3.^147^ Increased NRF2 expression levels in the OP model and its pharmacologically mediated activation appear to assume diametrically opposing roles in OP. Further mechanisms need to be studied.

## Osteoarthritis

### Role of NRF2 in OA

OA is one of the most prevalent chronic degenerative joint diseases. The main pathological features include synovial inflammation, articular cartilage degeneration of multiple joint structures, osteophyte formation, and subchondral bone sclerosis, resulting in severe pain and walking impairment. Symptomatic knee OA is present in 11% of men and 27% of women in the Asian population aged 65 years and over. The costs of OA are even roughly estimated to account for 1%–2.5% of the country's gross domestic product in many high-income countries.^148^ Existing treatments have limitations, and we need to identify more effective and safer methods to treat patients with OA.

**Figure 3:**
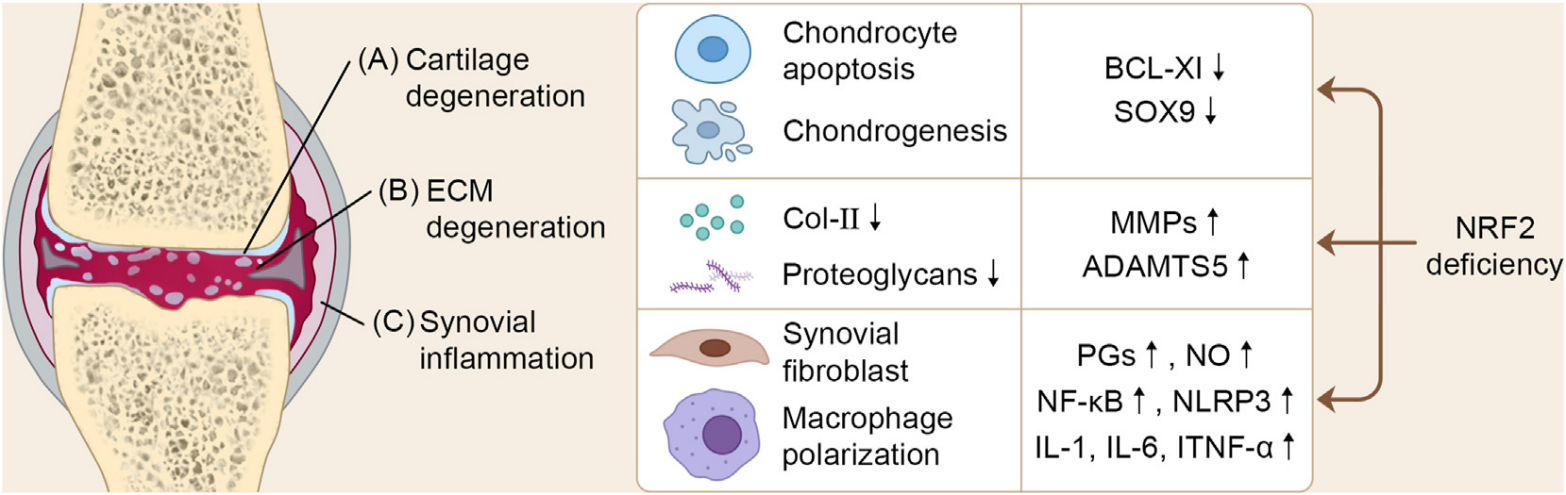
NRF2 deficiency contributes to the pathological processes of osteoarthritis (OA).

Recent findings suggest that excessive ROS production can lead to chondrocyte senescence/apoptosis (Fig. 3A), extracellular matrix (ECM) degradation (Fig. 3B), or synovial inflammation in OA (Fig. 3C).^149^

NRF2 function is decreased in OA, leading to up-regulation of NF-кB, COX-2, iNOS, and peroxynitrite expression, down-regulation of HO-1 expression, and increased severity of OA.^150^ Moderately activated NRF2 has been proven to activate the antioxidant defense system of chondrocytes and remove ROS. It also inhibits inflammation by affecting proinflammatory signaling pathways, thereby limiting bone resorption and cartilage degradation.^7^^,^^151^ In the early stages of OA, chondrocyte degradation enhances the OCs' breakdown process and accelerates bone resorption, which is attributed to favorable osteoclastogenesis with low NRF2/Keap1 ratios, as demonstrated in cases of NRF2 deficiency. On the other hand, in the later stages, it is more likely that the formation of bone spurs resulting from enhanced bone remodeling, as a compensatory mechanism, is related to osteoblastogenesis promoted by NRF2 overexpression.^7^ In addition, ROS is one of the key factors regulating OC differentiation. While ROS play an important role in the process of abnormal bone resorption in OA, NRF2 can activate the intracellular antioxidant system, inhibit OC differentiation caused by elevated ROS expression, and ultimately reduce the abnormal bone resorption in OA.^152^

### NRF2 regulates the pathogenesis of OA

The progression of OA is accompanied by multiple pathological processes, including oxidative system imbalance and chronic inflammation. It should be noted that NRF2 activity is due to ROS production induced by IL-1β.^153^ TNF-α, IL-1, IL-6, and prostaglandin E2 are factors mediating the cartilage degradation and inflammatory state in OA.^154^ Among them, TNF-α and IL-1β activate chondrocytes to produce ADAMTS5 and MMP-13, which degrades collagen type II (Col II) and proteoglycans, thereby leading to excessive degradation of ECM.^155^^,^^156^ IL-1β can activate MAPK pathways, such as p38, ERK, and NF-κB, and has a strong inhibitory impact on chondrogenesis, to aggravate the inflammatory state.^157^ NRF2 activation has been shown to induce the up-regulation of HO-1, which can be achieved by reducing the production of various inflammatory cytokines, including TNF-α, IL-1β, IL-6, and IL-18^158^ while inhibiting the pro-catabolic effect of IL-1β-induced MMP-1 and MMP-13 on ECM components.^159^ NRF2 comes into play in OA chondrocytes. In addition, it inhibits the NF-κB signaling pathway and reduces inflammatory mediators such as NO and prostaglandin E2.^155^ More detailed mechanisms of NRF2 in OA have also been explored recently. Yusuke Kubo and colleagues demonstrated the significance of SOX9 as a transcription factor for maintaining ECM homeostasis in chondrocytes and regulating chondrocyte differentiation by promoting the expression of Col II. In human chondrocytes, NRF2 has been found to act as a direct positive regulator of the SOX9 promoter by binding to ARE2, a site located in the proximal promoter region of SOX9. In NRF2 knockout mice, there was mild OA cartilage degeneration in aged mice, while knee cartilage in young and mature mice still looked normal.^68^ Autophagy and NRF2 coregulate chondrocyte differentiation. Recently, the research of Y. Horigom et al explained further mechanisms. Impaired autophagy reduces the number of mesenchymal cell-derived chondrocytes and causes growth delay in chondrocytes. Sequestosome 1 is up-regulated and binds to Keap1 in the mouse model of loss of autophagy to sustained activation of NRF2. It should be noted that sustained NRF2 activation may suppress the transition of mesenchymal cells to proliferating chondrocytes, but does not affect chondrogenesis starting from the round chondrocyte stage.^160^ Additionally, Zhou et al discovered that miR-146a is a target of NRF2, which is markedly increased in chondrocytes affected by OA. Overexpression of miR-146a suppresses NRF2 expression and worsens cartilage degeneration caused by OA. Activation of the NRF2/HO-1 pathway by agonists inhibits miR-146a and prevents cartilage degeneration.^161^ NRF2 deficiency is an important promoter of OA, while its activation prevents inflammation and the degradation of cartilage and ECM.

NRF2 protein levels have been reported to be decreased in human OA chondrocytes compared with normal chondrocytes,^162^ but this finding was contradicted by another study reporting that mRNA and protein expression of NRF2 was significantly up-regulated in cartilage from OA patients in another study. The research demonstrated that there was a significant increase in the expression levels of HO-1, NRF2, and NLRP3 inflammasome in the synovial tissue of patients diagnosed with OA. NLRP3 expression was up-regulated after NRF2 silencing in an *in vitro* inflammatory cell model.^163^ The difference in expression in the disease may be related to age, the degree of ROS accumulation, or the disease process. The increased NRF2 activity may be a compensatory factor, but the decreased NRF2 expression level caused by excessive oxidative stress and cell senescence undoubtedly accelerates the progression of OA.^25^

### NRF2 targets to protect cartilage and prevent ECM degradation

Plant-derived antioxidants are capable of regulating NRF2 expression and have been widely explored. These antioxidants have potent anti-inflammatory and antioxidant properties. They can remove ROS, suppress the expression of pro-oxidation genes, and up-regulate the expression of antioxidant genes. Wogonin increased NRF2 expression and activity and increased HO-1 expression, providing primary human chondrocytes with resistance to IL-1β-induced oxidative stress. Molecular assays showed that wogonin could disrupt the connection by directly inhibiting the NRF2 binding site in the Keap1 protein, which activated the ROS/ERK/NRF2/HO-1-NQO1-superoxide dismutase 2-glutamate-cysteine ligase catalytic subunit signaling axis to protect cartilage and ECM.^164^ By potentiating NRF2/HO-1, hyperoside restricts the activation of NF-κB. It down-regulates the expression of ADAMTS5 and MMP. Meanwhile, it promotes the up-regulation of SOX9, aggrecan, and Col II expression to alleviate IL-1β-induced ECM destruction. Mechanistically, it can exert an excessive anti-inflammatory effect by partially inhibiting the MAPK and PI3K/AKT/NF-κB signaling pathways. Moreover, hyperoside demonstrates an antiapoptotic impact by modulating the NRF2/ROS/BCL-xl axis.^165^

In addition to BCL-xl-associated cell death, NRF2 is also implicated in other forms of cell death. Zhou et al studied the efficacy of deferoxamine in OA, which has been used to inhibit ferroptosis as an iron-chelating agent in various degenerative disease models. Deferoxamine alleviated cytotoxicity in chondrocytes, eliminated the accumulation of ROS and lipid ROS, and promoted the expression of the NRF2 antioxidant system. Intra-articular injection of deferoxamine also enhanced Col II expression.^166^ Previous studies have demonstrated the crosstalk between NRF2 and autophagy in oxidative stress. Dong et al used CDDO-Im to up-regulate the pathway and increase the autophagy rate of chondrocytes treated with TNF-α. This significantly mitigated human chondrocyte apoptosis and ECM degradation.^167^ The association of NRF2 with immune cells may also be a treatment for OA. Recently, targeted therapy of macrophages in OA as the most abundant immune cells in synovial joints has attracted attention. The negative regulation of NF-κB and the expression of HO-1 by NRF2 may be the main promoting mechanisms that inhibit M1 polarization and promote M2 polarization.^168^ Lv et al found one of the targets, transient receptor potential vanilloid 1 (TRPV1), which is a cationic channel associated with pain perception and inflammation. TRPV1-induced Ca^2+^ influx promotes calcium/calmodulin-dependent protein kinase II phosphorylation, which increases nuclear translocation of NRF2 and ultimately leads to inhibition of M1 macrophage polarization.^169^ Taken together, antioxidant-induced NRF2 activation plays a significant anti-inflammatory role through its downstream genes and crosstalk with other pathways. It also prevents apoptosis and ECM degradation under oxidative stress.

## Intervertebral disc degeneration

### Role of NRF2 in IDD

IDD is a disease of imbalance between catabolic and anabolic processes in the intervertebral disc, resulting in excessive oxidative stress, loss of nucleus pulposus (NP) cells, changes in the composition of the ECM, and inflammation.^170^ Degeneration includes cartilaginous endplate (CEP) changes (sclerosis, defect, Modic changes, and osteophyte formation) and intervertebral disc changes (fibrosis, annulus fibrosus tear, water depletion, thinning, and annulus fibrosus myxoid degeneration). The incidence of IDD is linear with age.^171^ In addition to age, it is also related to many other factors, such as biomechanics and collagen quality. It is estimated that more than 90% of people over 50 have IDD, making it the predominant cause of chronic lower back pain and a leading contributor to disability.^172^ The current treatment of IDD is mainly to control disease progression and prevent patient disability. However, drug therapy and surgical treatment may cause complications with high costs but limited therapeutic effects.^173^

The pathological features of the disc include the loss of NP in the center and the replacement of nucleus lipocytes by cells with fibroblast-like phenotypes. Oxidative stress induces apoptosis of NP cells in the intervertebral disc. The inflammatory microenvironment and pyroptosis induced by the NLRP3 inflammasome may account for the loss.^174^ Anomalous acceleration of ECM degradation, exemplified by reducing Col II and proteoglycan, is a hallmark of IDD. This imbalance is primarily caused by an excessive degradation rate that surpasses the synthesis rate, leading to ECM deterioration.^175^ Oxidative stress promotes the degradation of the ECM by connecting with multiple critical signaling pathways in the NP cell, including the NRF2-ARE signaling, NF-κB signaling, and p38/MAPK signaling pathway.^176^^,^^177^ ROS also contribute to the aging, apoptosis, and ferroptosis of annulus fibrosus cells, which disrupts the characteristic architecture of the intervertebral disc.^178^^,^^179^ Moreover, the degeneration of the CEP on both sides of the intervertebral disc is also a crucial factor inducing IDD. Studies have revealed oxidative stress causes apoptosis, autophagy, and calcification of endplate chondrocytes, impeding the nutrient supply of the NP.^177^^,^^180^ It leads to the destruction of intervertebral disc homeostasis. A research study revealed a gradual down-regulation of NRF2 expression in human NP tissue samples with the progression of IDD.^181^ In conclusion, NRF2 deficiency was found to increase the progression of these pathological processes to varying degrees. Its decreased activity with age may be a key driver of disc degeneration.

### NRF2 targets protect the intervertebral disc microenvironment

**Figure 4:**
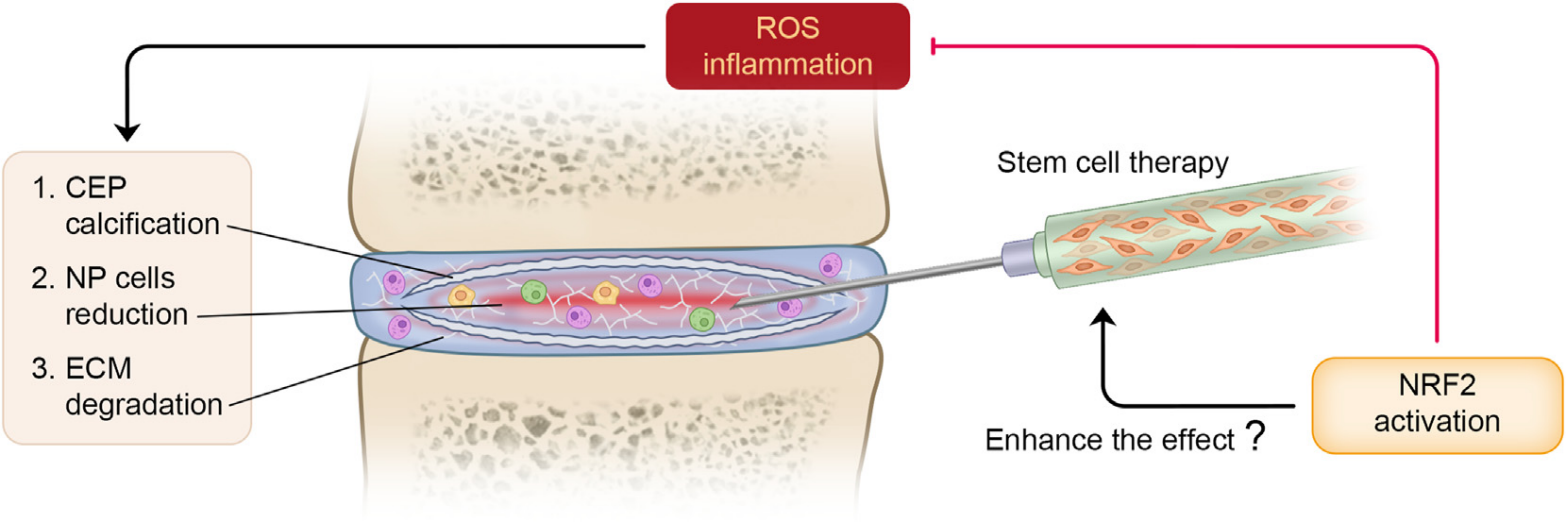
The activation of NRF2 rescues the microenvironment of intervertebral disc degeneration (IDD).

Targeted NRF2 antioxidant defense systems play a significant role in preventing IDD progression on account of their regulation of ROS and inflammatory cytokines (Fig. 4). Exogenous supplementation with MFG-E8 can save NP cells from ECM degradation and pyroptosis through the NRF2/TXNIP/NLRP3 axis.^182^ Cardamonin protects NP cells from IL-1β-induced catabolism and inflammation by inhibiting NF-κB and activating NRF2.^183^ ROS were eliminated, and mitochondrial dysfunction was alleviated in both experiments. Interestingly, a study by Hu et al demonstrated that tert-butylhydroquinone-mediated up-regulation of the NRF2/Sirt3 pathway restored autophagic flux disorder in an IDD model.^184^ Similarly, Tang et al also found that the presence of a p62-Keap1-NRF2 feedback loop drives ROS-induced autophagy in nucleus pulposus cells and prevents IDD.^181^

The calcification and degeneration of CEP hinder metabolite clearance and the transport of nutrients, which are critical initiating factors of intervertebral disk degeneration. Zuo et al used rapamycin to rescue cartilage endplate stem cells and avoid ECM degradation and calcification by activating autophagy/NRF2 signaling. TNF-α induces osteogenic differentiation, cell senescence, and oxidative stress of CEP stem cells. Autophagy induced by rapamycin has been shown to enhance the nuclear translocation and expression of NRF2, leading to an increase in the expression of antioxidant proteins and a reduction in ROS levels, which in turn alleviates cellular senescence. These effects of rapamycin-induced autophagy have been observed to enhance the chondrogenic differentiation potential of stem cells in the CEP.^185^ Kang et al found that polydatin could ameliorate CEP degeneration in a rat model and up-regulate NRF2 and Parkin levels in CEP-induced mitophagy to protect chondrocytes.^186^ Polydatin can not only effectively ameliorate CEP degeneration but also rescue NP cell senescence and excessive ECM destruction, which are induced by mitochondrial homeostasis via the NRF2/HO-1 pathway.^186^^,^^187^

NRF2 activation resists oxidative stress and inflammation in IDD. Enhancing NRF2-dependent autophagy signal transduction is an effective way to protect NP cells and endplate chondrocytes.

### NRF2 in cell therapy in IDD

Maintaining or increasing the number of viable cells in degenerative intervertebral discs and altering the balance between synthesis and degradation to maintain or rebuild the function of ECM are emerging therapeutic strategies. Li and Peng found that cell therapy by injecting exogenous cells into the intervertebral disc to repair degenerative intervertebral discs is emerging as a promising approach. Cell therapy involves delivering live cells to the nucleus pulposus, annulus fibrosus, or systemic application, alone or combined with a biomaterial scaffold and carrier to refill and repair the degenerative intervertebral disc or at least modulate the degenerative microenvironment.^188^ Many preclinical studies and clinical trials have shown that cell therapy can promote cell proliferation and anabolic activity to increase the possibility of restoring intervertebral disc homeostasis and can also relieve discogenic pain through immune regulation and inhibition of inflammation.^189^ The obstacle to this clinical application of therapy is maintaining the survival of resident and exogenous cells, which still need to face ischemia, hypoxia, high-pressure load, and even lack of nutrition after CEP calcification. In previous research, an excellent therapeutic effect was found by transplanting NRF2-overexpressing neural progenitor cells into the mouse brain. The differentiation and migration of neural progenitor cells from the injection site were evaluated through the transplantation of neural progenitor cells overexpressing NRF2 into the striatum of a mouse model with neurodegenerative conditions. The results revealed a remarkable reduction in lesion volume by approximately four-fold, indicating an unexpected outcome.^190^ According to Zhang et al, pretreatment of nucleus pulposus mesenchymal stem cells with 75 μM H_2_O_2_ resulted in significantly increased cell proliferation, enhanced ability to counteract oxidative stress, and reduced apoptosis in the context of IDD treatment. The study also revealed a remarkable up-regulation of NRF2 expression following H_2_O_2_ treatment. The results were also confirmed *in vivo*.^191^ Regulating NRF2 in cell therapy may have unexpected effects, such as promoting cell survival, maintaining stem cell differentiation ability, and resisting inflammation.

## Conclusion and perspectives

The expression of NRF2 has been shown to play a pivotal role in human development and the pathogenesis of numerous AMSDs, including sarcopenia, OP, OA, and IDD. These diseases are closely linked to cellular metabolism and oxidative stress, which are regulated by the downstream effects of NRF2. Chronic inflammation, cellular dysfunction, and apoptosis can be triggered by factors such as aging and oxidative stress, and current treatments for these disorders are inadequate. As a vital antioxidant transcription factor, NRF2 safeguards cells from the damaging effects of oxidative stress. However, the loss of its function due to the up-regulation of negative regulatory factors or epigenetic inhibition can lead to increased oxidative products, which is a crucial aspect of degenerative diseases. To combat these effects, various antioxidants such as natural product-derived small molecules, bioactive compounds, and specific noncoding RNAs have been demonstrated to activate NRF2 signaling, providing promising therapeutic avenues for alleviating and preventing the progression of these degenerative diseases.

NRF2 activation contributes to maintaining the structure and function of musculoskeletal and intervertebral disc integrity by suppressing the inflammatory response, cell senescence, apoptosis, and ECM degradation, making it a potential therapeutic strategy for AMSDs. Physical exercise has shown therapeutic effects on OP and sarcopenia, in which the NRF2 pathway may play a crucial role. Despite the significant progress in research on NRF2 and AMSDs, there are still challenges to be addressed in terms of mechanistic research and clinical translation.

(i) Regulating the proliferation and differentiation of chondrocytes, OBs, OCs, and stem cells by controlling NRF2 signaling also shows the feasibility of its treatment for degenerative diseases. It is essential to consider that NRF2 is involved in numerous biological functions and exhibits specificity within various cell types and tissues. While cell therapy may be a suitable option, further studies are required to achieve more precise regulation.
(ii) Mitochondria, as a primary source of intracellular ROS, are closely related to oxidative stress, and the regulation of mitochondrial function by NRF2 signaling may be an area of future interest.
(iii) Additionally, the crosstalk between NRF2 and critical signaling pathways or mechanisms, such as ferroptosis, remains controversial. While most studies have shown that NRF2 activation mitigates lipid peroxidation and prevents ferroptosis, it has been noted that NRF2-mediated up-regulation of HO-1 impairs iron/heme homeostasis and induces ferroptosis. Further systematic studies are still required to explore the multiple interrelated mechanisms involved in individual diseases.
(iv) Although NRF2 shows immense potential in biological experiments, there is still a long journey ahead to translate it into clinical therapy. The variation in NRF2 expression levels among individuals in the population may be the next area of focus. Factors such as genetics, age, diet, and disease status influence the extent of individual NRF2 activity, which is crucial for the clinical translation and drug development of this target.
(v) While various pharmacological NRF2 activators have demonstrated benefits in preventing disease progression by resisting ROS, only a few of them, such as dimethyl fumarate and sulforaphane, have been approved for treating specific conditions like multiple sclerosis and diseases associated with cell damage. More research is needed to identify the underlying molecular mechanisms and conduct clinical trials to repurpose or develop drugs targeting NRF2 for indications such as AMSDs, while also being cautious to avoid treatment resistance and its excessive activation that may cause disease.

## Conflict of interests

The authors declare that they have no competing interests.

## Funding

This work was supported by the National Key R&D Program of China (No. 2019YFA0111900), the 10.13039/501100001809National Natural Science Foundation of China (No. 82072506, 92268115, 82071970, 81971775), the Science Fund for Distinguished Young Scholars of Hubei Province (No. 2023AFA109), the Science and Technology Innovation Program of Hunan Province (No. 2021RC3025), the Independent Exploration and Innovation Project for Postgraduate Students of Central South University (China) (No. 2022ZZTS0268), 10.13039/501100010083Hunan Provincial Innovation Foundation for Postgraduate (No. CX20220350), the Science and Technology Project of Jianghan University (No. 2022SXZX25), and the Science and Technology Innovation Project of Jianghan University (China) (No. 2021kjzx008).
