# Supplementary material for: NRF2 in age-related musculoskeletal diseases: Role and treatment prospects
Source: Genes Dis. 2023 Nov 27;11(6):101180. doi: 10.1016/j.gendis.2023.101180 (PMC11400624; doi:10.1016/j.gendis.2023.101180)
Supplement: Multimedia component 1 [file mmc1.docx]

NRF2 (Nuclear Factor-Erythroid Factor 2-related factor 2) is a transcription factor encoded by the NFE2L2 gene, which initiates protective responses within cells by regulating the expression of multiple genes. In recent years, NRF2 has been found to play a widespread role in cellular activities, including cell metabolism, antioxidant signaling, protein homeostasis, and iron metabolism. Its expression levels influence various cellular processes, including oxidative stress, inflammation, mitochondrial dysfunction, cellular aging, apoptosis, ferroptosis, proliferation, and differentiation. Research into age-related diseases has revealed that NRF2 is closely associated with inflammation, increased reactive oxygen species (ROS), mitochondrial dysfunction, and cell apoptosis. Animal models with NRF2 deficiencies have shown a higher susceptibility to age-related diseases such as heart disease, musculoskeletal loss, and cancer. This has made NRF2 a current focal point in clinical research and translational medicine.

Professor Li Yusheng's team at Central South University has published a review in this journal titled "*NRF2 in Age-related Musculoskeletal Diseases: Role and Treatment Prospects*," summarizing the latest advancements in research on the pathogenic mechanisms of NRF2 in age-related musculoskeletal diseases and exploring its potential as a target for drug development or cell therapy.


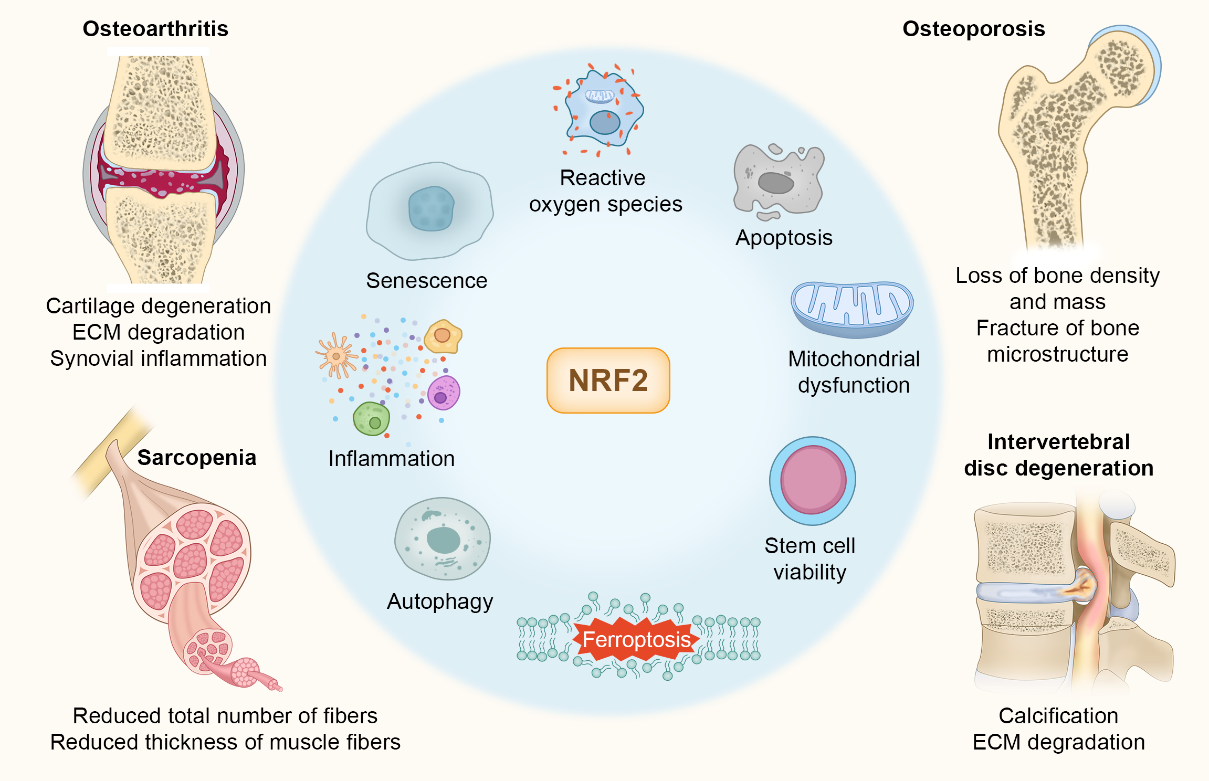


Figure 1 Association of NRF2 with Age-Related Musculoskeletal Diseases (Original: Graphic Abstract)

Musculoskeletal diseases are defined as injuries to muscles, bones, joints, and adjacent connective tissues, resulting in short-term or lifelong limitations in physical function and mobility. Statistics indicate that approximately 1.71 billion people are affected by musculoskeletal diseases. Pathological changes in muscle, bone, and intervertebral discs can lead to age-related musculoskeletal diseases, including muscle wasting, osteoporosis, osteoarthritis, and intervertebral disc degeneration. This review, using NRF2 as a focal point, extensively summarizes its role and the current state of research on translational treatments for these four musculoskeletal diseases. It highlights the multifaceted roles of NRF2 in mitochondrial dysfunction, chronic inflammation, oxidative stress, and ferroptosis in different diseases, as well as its potential as a target for drug development and cell therapy.

The expression of NRF2 has been shown to play a pivotal role in human development and the pathogenesis of numerous AMSDs, including sarcopenia, OP, OA, and IDD. These diseases are closely linked to cellular metabolism and oxidative stress, which are regulated by the downstream effects of NRF2. Chronic inflammation, cellular dysfunction, and apoptosis can be triggered by factors such as aging and oxidative stress, and current treatments for these disorders are inadequate. As a vital antioxidant transcription factor, NRF2 safeguards cells from the damaging effects of oxidative stress. However, the loss of its function due to upregulation of negative regulatory factors or epigenetic inhibition can lead to increased oxidative products, which is a crucial aspect of degenerative diseases. To combat these effects, various antioxidants such as natural product-derived small molecules, bioactive compounds, and specific noncoding RNAs have been demonstrated to activate NRF2 signaling, providing promising therapeutic avenues for alleviating and preventing the progression of these degenerative diseases.

NRF2 activation contributes to maintaining the structure and function of musculoskeletal and intervertebral disc integrity by suppressing the inflammatory response, cell senescence, apoptosis, and ECM degradation, making it a potential therapeutic strategy for AMSDs. Physical exercise has shown therapeutic effects on OP and sarcopenia, in which the NRF2 pathway may play a crucial role. Despite the significant progress in research on NRF2 and AMSDs, there are still challenges to be addressed in terms of mechanistic research and clinical translation:

a. Regulating the proliferation and differentiation of chondrocytes, OBs, OCs, and stem cells by controlling NRF2 signaling also shows the feasibility of its treatment for degenerative diseases. It is essential to consider that NRF2 is involved in numerous biological functions and exhibits specificity within various cell types and tissues. While cell therapy may be a suitable option, further studies are required to achieve more precise regulation.

b. Mitochondria, as a primary source of intracellular ROS, are closely related to oxidative stress, and the regulation of mitochondrial function by NRF2 signaling may be an area of future interest.

c. Additionally, the crosstalk between NRF2 and critical signaling pathways or mechanisms, such as ferroptosis, remains controversial. While most studies have shown that NRF2 activation mitigates lipid peroxidation and prevents ferroptosis, it has also been noted that NRF2-mediated upregulation of HO-1 impairs iron/heme homeostasis and induces ferroptosis. Further systematic studies are still required to explore the multiple interrelated mechanisms involved in individual diseases.

d. Although NRF2 shows immense potential in biological experiments, there is still a long journey ahead to translate it into clinical therapy. The variation in NRF2 expression levels among individuals in the population may be the next area of focus. Factors such as genetics, age, diet, and disease status influence the extent of individual NRF2 activity, which is crucial for the clinical translation and drug development of this target.

e. While various pharmacological NRF2 activators have demonstrated benefits in preventing disease progression by resisting ROS, only a few of them, such as dimethyl fumarate and sulforaphane, have been approved for treating specific conditions like multiple sclerosis and diseases associated with cell damage. More research is needed to identify the underlying molecular mechanisms and conduct clinical trials to repurpose or develop drugs targeting NRF2 for indications such as AMSD, while also being cautious to avoid treatment resistance and its excessive activation that may cause disease.
